# Preventive HIV Vaccines-Leveraging on Lessons from the Past to Pave the Way Forward

**DOI:** 10.3390/vaccines9091001

**Published:** 2021-09-08

**Authors:** Parveen Sobia, Derseree Archary

**Affiliations:** 1 Centre for the AIDS Programme of Research in South Africa (CAPRISA), Nelson Mandela School of Medicine, University of KwaZulu-Natal, Durban 4001, South Africa; parveen.sobia@caprisa.org; 2 Department of Medical Microbiology, University of KwaZulu-Natal, Durban 4001, South Africa

**Keywords:** vaccines, HIV, antibodies, mucosal, immune responses

## Abstract

Almost four decades on, since the 1980’s, with hundreds of HIV vaccine candidates tested in both non-human primates and humans, and several HIV vaccines trials later, an efficacious HIV vaccine continues to evade us. The enormous worldwide genetic diversity of HIV, combined with HIV’s inherent recombination and high mutation rates, has hampered the development of an effective vaccine. Despite the advent of antiretrovirals as pre-exposure prophylaxis and preventative treatment, which have shown to be effective, HIV infections continue to proliferate, highlighting the great need for a vaccine. Here, we provide a brief history for the HIV vaccine field, with the most recent disappointments and advancements. We also provide an update on current passive immunity trials, testing proof of the concept of the most clinically advanced broadly neutralizing monoclonal antibodies for HIV prevention. Finally, we include mucosal immunity, the importance of vaccine-elicited immune responses and the challenges thereof in the most vulnerable environment–the female genital tract and the rectal surfaces of the gastrointestinal tract for heterosexual and men who have sex with men transmissions, respectively.

## 1. Introduction

To date, the human immunodeficiency virus (HIV) has claimed more than 32.7 million lives and globally, there is an estimated 38 million people are living with the disease [1]. Approximately 67% of these infected individuals are currently on antiretroviral (ARV) treatment [1]. Although several human clinical trials testing the effectiveness of ARVs in pre-exposure prophylaxis (PrEP) and treatment as prevention (TAsP) were highly successful, HIV infections continue to proliferate [2,3,4]. With about 4500 daily new infections globally, HIV remains a formidable public health challenge [1]. An efficacious HIV vaccine is therefore essential to prevent the cycle of new infections. The HIV vaccine field as a whole has been fraught with disappointment, modest success, and more recently, disappointment again, forcing the field back to the drawing board, and hinging on results from ongoing trials for a glimmer of hope. In this review, we provide an update on the HIV vaccine field, the most recent obstacles, and advancements for their development. We also elaborate on the evolution of passive immunization trials testing the use of HIV broadly neutralizing antibodies (BnAbs) in clinical management and treatment of HIV disease. We present the most recent data from clinical trials, testing the safety of new generation, potent, BnAbs as a putative HIV prevention option. Finally, we highlight the distinct immune responses in the predominant sites for HIV entry during sexual intercourse—the genital and the rectal mucosal surfaces and how critical the heterogeneity of immune responses is, as well as the challenges in developing an efficacious HIV vaccine.

## 2. HIV Vaccine Trials-Past and Present

In the late 1980’s, a fundamentally empiric approach to elicit the production of antibodies (a correlate of protection found in vaccination against other infectious diseases) [5] prompted the HIV vaccine field to test protein immunogens directed against the outer envelope protein of HIV. Recombinant envelope (Env) subunit gp160 was tested in 1988 in 72 healthy adults in Canada, and although this vaccine was deemed safe and generated some homologous neutralizing antibodies, an immunogen dose escalated design in some individuals. The vaccine-elicited antibodies, however, did not confer protection, and the vaccine was not pursued [6,7]. In fact, when this recombinant gp160 vaccine was tested in early-stage HIV infected individuals, there was no therapeutic benefit, no significant impact on CD4 T-cell counts, and no difference in viral loads between the active vaccine arm compared to the placebo [8]. Subsequent to this, in the 1990’s, recombinant gp120 (rgp120) protein-based vaccines were tested in two Phase III randomized, double-blind, placebo-controlled efficacy trials (Table 1).

In 1998 the VAX004 (AIDS VAXB/B) and in 1999 the VAX003 (AIDS VAXB/E) trials started using bivalent rgp120 with alum. The VAX004 trial enrolled 5403 participants who were men-who have sex with men (MSM) and women at high risk for HIV infection in North America and the Netherlands [9]. The trial tested the efficacy of the vaccine containing two rgp120 HIV-1 envelope antigens derived from two different subtype B strains [9]. The VAX003 trial recruited 2546 participants consisting of intravenous injection drug users (IDU) in Thailand, vaccinated with two rgp120 HIV-1 envelope antigens derived CXCR4-dependent subtype B (MN) and CCR5 dependent subtype AE (A244) [10]. In 2004, however, both Phase III trials showed failure in conferring protection despite the generation of anti-gp120 antibodies. These two vaccine trial failures, using recombinant gp120, highlighted that monomeric gp120 may be inappropriate as a vaccine immunogen stimulating protective antibody responses. The failure of monomeric gp120 to elicit protective humoral immunity, despite the generation of neutralizing antibodies in human clinical trials, highlights the discordance between the antibody responses generated against lab-adapted HIV strains as opposed to circulating primary viral isolates. These data also highlight how crucial the quaternary conformation of gp120 on primary viruses are to the immune system and that the vaccine monomeric gp120 may have baited the immune system with otherwise poorly presented epitopes, rendering the ensuing antibody responses weakly neutralizing or non-neutralizing [11].

After these vaccine failures, the field moved towards T cell-based vaccines, using live recombinant viral vectors, such as pox and replication deficient adenovirus vectors [12,13,14]. In 2004, the multi-center HVTN502 (STEP) trial tested the adenovirus vectored vaccine. The MRK-adenovirus 5 vector (Ad5), containing *gag*/*pol*/*nef* genes in a Phase IIb trial, was tested in 3000 high-risk healthy women and MSM [15]. This trial was premised on several studies that showed that T-cell-mediated immunity and function ameliorated the disease [16,17,18,19,20], and mounting evidence for cytotoxic T-cell responses driving viral control [21,22,23]. Indeed, preclinical animal studies further supported the notion that a lack of cytotoxic T-cells or robust T-cell-mediated protection was a correlate for increased risk of HIV and supported the further testing of this vectored vaccine in human clinical trials [24,25,26]. This trial was prematurely halted in September of 2007 due to futility and indications of increased infections among vaccinees due to higher pre-existing adenovirus-5 (Ad-5) antibodies. To explain this significant observation, several hypotheses were put forward. The first was that the vaccinees with pre-existing Ad-5 antibodies had increased Ad-5 specific CD4 T-cells that rapidly expanded upon vaccination. This rapidly expanded and activated Ad-5 specific CD4 T-cell subset may have homed to vulnerable mucosal sites which then served as targets for HIV infection [27]. The second hypothesis, supported by in vitro studies, was that pre-existing Ad5-specific neutralizing antibodies modified HIV risk through immune complex formation following vaccination, with subsequent changes in inflammatory profiles that may have led to greater immune cell activation [28]. The third hypothesis put forward suggested that increased infections among uncircumcised men in HVTN502 could be attributed to Ad5-specific neutralizing antibodies that augmented immune complex formation following vaccination, which then stimulated dendritic cell maturation and inflammation. Unfortunately, the follow-on HVTN 503 (Phambili) trial in South Africa, which also tested the MRK-Ad5 clade B *gag*/*pol*/*nef*, suffered the same fate due to futility just eight months after initiation in 2007, and indications of increased infections were also observed in male vaccinees [15]. Despite these setbacks, the HVTN502 trial provided critical data and cautioned that T-cell activation may be used as an immune correlate for increased HIV risk. In particular, induction of non-specific IFN-γ from CD4 T-cells pre-infection after the initial vaccination significantly modified HIV risk in those vaccinees [29]. Immunology studies on individuals given the Ad5 vectored vaccine from HVTN502 did not show long-term changes in the activation or homing of mucosal CD4 or CD8 T-cells, regardless of baseline pre-existing Ad5 antibodies [30]. The HVTN505 phase IIb randomized, placebo-controlled, efficacy trial initiated in 2009. A DNA-primed vaccine was tested in men or transgender women who have sex with men. The prime-boost regimen was given as three vaccinations with DNA encoding HIV clade B *gag*/*pol*/*nef*/*env* from HIV clades A, B and C followed by an Ad5 vector-based vaccine encoding clade B *gag*/*pol*/*env* from clades A, B, and C [31,32,33]. This trial was also prematurely halted due to increased HIV infections in the vaccinees receiving active product compared to the placebo [34]. Further research regarding the immune responses revealed that this specific prime-boost approach elicited non-neutralizing gp41 antibodies [35] and a weak antibody response to Env V1V2 on gp120 [36]. In addition to being unable to neutralize HIV, these gp41 antibodies were also cross-reactive to bacterial proteins such as those found in *Escherichia coli*, a commensal bacterium which naturally occurs in the gut microbiome. As such, it was posited that some of the pre-existing gp41 antibodies primed by these gut microbial proteins may have diverted any protective responses [35]. All three of these trials—HVTN502, HVTN503 and HVTN505–were stopped due to increased infections in the vaccine arm, creating huge setbacks for the field [37].

Currently, there are two ongoing phase IIb trials. The HVTN705/Imbokodo trial is testing the efficacy of a heterologous prime/boost vaccine regimen of Ad26.Mos4.HIV and aluminum phosphate-adjuvanted clade C gp140 in sexually active women from multiple sites in sub-Saharan Africa [38]. A complementary ongoing vaccine trial, HVTN706 or Mosaico, is testing a similar heterologous regimen of Ad26.Mos4.HIV and adjuvanted aluminum phosphate-Clade C gp140 and Mosaic gp140 in healthy transgender people and MSM in North America and Europe. The difference between these two trials is that Mosaico uses gp140 immunogens from different HIV strains to account for diversity and induce diverse immune responses, while Imbokodo includes a clade C gp140 immunogen. These two trials were premised on and supported by data from a multi-center phase I/IIa clinical trial and a non-human primate (NHP) study—APPROACH [38], and in two others in Phase I/IIa trials—TRAVERSE [39], and ASCENT. Preliminary findings from ASCENT presented at the International AIDS Society (IAS) Conference in 2019 [40] and published data from TRAVERSE showed increased magnitude of cross-clade vaccine induced binding antibody responses, increased Env-specific CD4 T-cell responses and no reported interference by pre-existing antibodies to Ad26 [39]. Data from the Imbokodo and Mosaico trials are expected from 2022 onwards. These remain the only two HIV vaccine phase IIb trials in the field and puts into perspective the challenges in developing an efficacious vaccine.

Reconciling the lessons from these past vaccine failures to pave the way forward, the vectored vaccine approaches definitely brings into perspective the importance of fully defining the effect of pre-existing immunity. This is due to the limitations of extrapolating immune correlates of protection or risk found in non-human primate models to humans, and the importance of genital mucosal sampling in understanding the mechanisms of infection in relation to vaccine-induced immune responses at sexual surfaces. However, going forward, there are cytomegalovirus (CMV) vectors that remain attractive options for vaccine vectors since their infection could trigger broad and robust humoral and cellular immunity [41,42]. Much of the immune correlates of protection are guided by evidence from NHP models using Rhesus cytomegalovirus strain expressing simian immunodeficiency virus (SIV) proteins (RhCMV68-1/SIV). Not only did RhCMV68-1/SIV vaccination elicit immunity that provided the early control of infection and persistent protection for the majority of the rhesus macaques when challenged with highly pathogenic SIVmac239 virus [43], but it also helped clear SIV infection despite viral dissemination [44]. These data compellingly showed that robust effector memory T-cells were effective against SIV infection rather than antibodies [45], and the potential for exploiting CMV vectorized delivery of HIV proteins may be promising. However, relying on T-cell-mediated immunity alone (similarly to HIVTN502/HVTN503) or B-cell-mediated antibodies (similarly to Vax003/Vax004) may not be enough to prevent HIV, and that both arms of adaptive immunity need to be stimulated and work in concert for elicitation of effective and protective immunity.

**Table 1:** Major HIV vaccine trials past and current.

Name of Vaccine Trial	Vaccine (with or without Vector)	Phase of Trial	Number of Trial Participants and Trial Period	Virus and Immune Correlates Observed for Reduced or Increased Risk	Reasons for Trial Termination or Discontinuation
AIDSVAX B/E (VAX003)	Two rgp120 envs-Clade B and CRF01_AE Env antigens in alum	III	2546 1999–2003	Gp120 antibodies	No efficacy
AIDS VAX B/B (VAX004)	Clade B recombinant Env recombinant gp120 (rgp120) in alum	III	5417 1998–2003	Neutralizing gp120 antibodies and CD4 blocking antibodies	No efficacy
HVTN502 (STEP)	Merck Ad5 HIV-1 *gag*/*pol*/*nef*	IIb	3000 2004–2007	Pre-existing Ad5 antibodies, ex vivo IFN-γ and interleukin-2 secretion from CD4 and CD8 T cells [29]	Trial halted-No Efficacy-increased infections in vaccine group [14,34]
HVTN503 (Phambili)	Merck Ad5 Clade B *gag*/*pol*/*nef*	IIb	801 of 3000 February–September 2007	T cell-mediated virus pressure on infecting virus premised by vaccine proteins	Trial halted-No efficacy-unblinded analyses-increased infections-male vaccine group
HVTN505	DNA vaccine with Clade B *gag*/*pol*/*nef*, & recombinant Ad5 with Clade B *gag*/*pol* & Clades A/B/C *env*	III	2504 2009–2017	Virus Env mutations on CD4 binding site likely T cell-mediated Gp70 V1V2 antibodies were lower in HVTN 505 [46] than in RV144 [47]. The response to V3 CRF01_AE also inversely correlated with the risk of HIV infection in vaccine recipients with lower levels of Env-specific plasma IgA and neutralizing antibodies	Trial Halted Increased infections in vaccine group-no efficacy. MIT analyses showed no differences [36]
HVTN705 (Imbokodo)	Heterologous Prime/Boost Regimen Ad26.Mos4.HIV and with aluminum adjuvanted-clade C Env gp140	IIb	2637 2017–2022	Trial Ongoing	Trial Ongoing
HVTN706 (Mosaico)	Heterologous Regimen Ad26.Mos4.HIV and adjuvanted aluminum phosphate-clade C gp140 and Mosaic gp140	IIb	3800 2019–2024	Trial Ongoing	Trial Ongoing

HVTN—HIV Vaccine Trials Network, Env—envelope, MIT—Modified intention-to-treat.

In 2003, the RV144 trial, which tested the ALVAC-HIV canarypox vectored vaccine plus the AIDSVAX B/E recombinant gp120 protein boosts, were initiated in more than 16,400 individuals in Thailand (Table 2) [48]. In 2009, the modified intention-to-treat analysis from the RV144 trial demonstrated a modest 31.2% efficacy. This trial led to the discovery of several correlates for reduced HIV infection risk. These included increased circulating Env V1V2 binding IgG antibodies in vaccinees that were also later shown to demonstrate non-neutralizing antibody effector functions such as enhanced antibody dependent cellular cytotoxicity (ADCC), phagocytosis, and also complement activation [49,50,51]. These findings of Fc-mediated viral control were also supported in NHP and other human studies [51,52,53,54,55]. In contrast, increased circulating Env V1V2 IgA in RV144 correlated with reduced protection and the mechanism postulated was that the Env V1V2 IgA isotype may have blocked or inhibited the Env V1V2 IgG from binding to the virus [56,57]. The original trial data prompted a slew of subsequent trials, testing whether extended protein boosting or different adjuvants improved their immunogenicity and durability of immune responses, ultimately hoping to achieve an improvement in vaccine efficacy [58].

To recapitulate the findings and improve the efficacy of RV144, a repeat of the RV144 trial was conducted in southern Africa (HVTN702 or Uhmabo). HVTN702, was a large randomized, controlled, double-blind study that was conducted at 14 sites. HVTN702, conducted in South Africa from 2016 to 2020, had several modifications from the original RV144 vaccine. These included the use of adjuvant MF59 and bivalent subtype C gp120/MF59 in a prime-boost vaccine regimen of ALVAC-HIV (vCP2438) (listed in Table 2). Prior to the initiation of HVTN702, HVTN100 was conducted in South Africa. The recent results of the HVTN100 immunogenicity studies showed higher rates of IgG3 responses to Env gp120, significantly higher CD4+ T-cell responses and gp120 binding antibody responses, compared to those of RV144. In addition, there was increased antibody durability after the 12-month vaccination period, with the subtype C protein-pox combination, compared to that of six months [59]. Specifically, these promising data from HVTN 100 were used to advance phase IIb/III clinical trial testing-HVTN702. Despite the promise that HVTN702 offered from its predecessor study, HVTN100, this trial also suffered a disappointing result with more infections in the vaccine arm than those in the placebo. HVTN702 was stopped due to futility and investigations are ongoing to understand why the trial failed to show efficacy despite the increased antibody durability and cellular responses from the preceding HVTN100. Once again, the HIV vaccine field suffered major setbacks and currently there are only two ongoing phase IIb trials, HVTN705 and HVTN706.

DNA vaccines were also explored as an alternative to stimulate humoral and cellular immune responses. Besides being relatively easy to generate, the added advantage of the DNA vaccines is that specific genes encoding specific proteins can be included. Once inside, the cells can manufacture these antigens that are conformationally apt to subsequently stimulate the production of specific immune responses [60,61]. DNA vaccines expressing HIV genes have been investigated in HIV-infected and healthy people for their safety and ability to prime or boost virus-specific immune responses [62,63,64]. Although DNA vaccines were safe, they too failed to elicit robust levels of T-cells or antibody responses. To boost the immune responses during DNA vaccination, the use of molecular adjuvants [65,66] for the elicitation of specific cytokines is also being explored. However, various routes of DNA vaccine delivery systems from chemical to physical (gene-gun, biojector, electroporation and intramuscular injection) are being investigated for optimal delivery which affect ensuing immune responses [67,68,69,70,71,72,73,74,75]. For now, DNA vaccines remain further from the clinical trial pipeline despite incremental advances in the field.

Very recently, the advent of mRNA vaccine technology and the high efficacies against severe COVID-19 disease reported from Pfizer Biontech and Moderna mRNA vaccine trials have [76,77] also paved the pathway for candidate HIV vaccines exploiting the same technology. Particularly significant is the elicitation and detection of SARS-CoV-2 specific antibodies, the immune correlate of protection, in the saliva and in the sera of mRNA vaccinated individuals [78] which could plausibly be extrapolated to the types of mucosal and circulating immune responses that may be crucial to vaccine efficacy for prevention by an mRNA HIV vaccine. However, the current breakthrough SARS-CoV-2 infections with the delta variants in fully vaccinated populations and the waning SARS-CoV-2-specific antibodies remind us that even these newer mRNA vaccines may not confer lasting immunity against varied strains and require booster vaccines. These data argue that mRNA HIV candidate vaccines may also require the same in order to achieve efficacy.

**Table 2:** RV144 vaccines and all subsequent vaccine testing based on the original RV144 trial data.

Name of Vaccine Trial	Vaccine Used	Phase of Trial	Number of Trial Participants and Trial Period	Immune Correlates Observed for Reduced or Increased Risk	Endpoint of Trial
* RV144	ALVAC HIV-AIDSVAX B/E	III	16,402 2003–2006	Binding antibodies High Env V1V2 IgG effecting ADCC V1V2 IgG3-elicited ADCC [49,50,79]	Efficacy: 31.2% efficacy at 42 months [48] 60% efficacy at 12 months
RV305	ALVAC HIV (vCP1521)/Extended boosting AIDSVAX B/E at 0 days and 6 months-must have participated in the RV144 Trial 6–8 years earlier	II-3 groups	162 2012–2017	Higher magnitude humoral antibodies and CD4 T cell responses than RV144 but magnitude did not increase with subsequent boosting [80]	Immunogenicity
RV306	ALVAC HIV (vCP1521) at 0 and 1 months/ ALVAC-HIV in combination with AIDSVAX B/E at months 3 and 6-Boosting took place at months 12, 15, or 18 by dual immunization-vector & protein or protein alone at 12-months	II-4 groups	360 2013–2017	IgG titres against V1V2 epitopes matched to the ALVAC-HIV envelope insert. Groups boosted at 18 rather than 15 months had higher, and broader IgG responses to gp120 than gp70V1V2. IgG:IgA ratios increased [81]	Immunogenicity [81]
#HVTN100	ALVAC HIV (vCP2438) alone at months 0 and 1/bivalent subtype C gp120 adjuvanted either MF59 or aluminum hydroxide at months 3, 6, and 12	I/II	252 2015–2017	Booster of subtype C pox-protein vaccines at month 12 improves the durability of vaccine-induced immunity-Antibody and cellular response rates were higher after the 12-month booster than after the 6-month vaccination	Immunogenicity to extend vaccine-induced immune responses [59]
HVTN702 (Uhambo)	ALVAC-HIV (vCP2438) and a gp120 subtype C gene Plus Clade B *gag*/*pro* genes plus two clade C gp120 Env proteins (TV1 and 1086) subunit vaccine with MF-59 adjuvant at 12 and 18 months	IIb/III	5407 2016–2020	Still under investigation	Efficacy-Terminated due to futility

Since 1987, despite the clinical testing of 30 vaccine candidates, in 60 Phase I/II studies, only 6 HIV phase III vaccine trials were completed, and 2 of them have produced disappointing findings, resulting in huge setbacks for HIV prevention [82]. Alternative strategies especially in the absence of an effective HIV vaccine, such as passive immunity, have reinvigorated interest in using HIV monoclonal antibodies as proof of concept for HIV prevention.

## 3. Passive Immunity

Naturally, passive transfer of antibodies in mammals in utero and through breastmilk from mothers to their newborn young constitutes among the first protective immunity conferred. Subsequently, this concept of using passive immunity in medicine was adapted and copied from nature, in order to prevent infections pre- and post-pathogen exposure. The most notable examples are Hepatitis B and Varicella zoster gamma globulin administration to newborns exposed to Hepatitis B from their infected mothers and individuals exposed to chickenpox [83]. In HIV mother-child-transmission studies, HIV infected mothers with high-titred HIV-specific antibodies were less likely to transmit HIV and infect their babies, suggesting that these antibodies may have at least protected babies from in utero infection [84,85,86]. In healthy HIV-infected individuals, high-titred neutralizing antibodies or HIV-specific binding antibodies correlated with protection against disease progression and pathogenesis. Early passive immunity studies in the 1990’s, using HIV-immunoglobulins (HIVIG) in HIV-infected individuals, also showed some promise with transient declines in viral loads and some “vaccinal” effects [87,88] of increased autologous antibody titres [89,90,91,92,93]. However, due to the short half-lives of HIVIG, patients experienced a viral load rebound. As a therapeutic alternative, other studies did not recapitulate findings of disease amelioration after treatment with HIVIG. The mixed results from these passive immunity studies left the field rather discouraged for the use of antibodies as a therapeutic alternative, especially since there were limited antiretroviral drugs and classes of drug options available, as well as disappointing results from HIV vaccine trials. The discovery of new and more potent HIV monoclonal antibodies, referred to as BnAbs, have the ability to neutralize a wide variety of primary and reference strain viruses, thus giving momentum once again to their use for passive immunity. However, the staccato discovery of these BnAbs was in part due to the prevailing technologies at the time that were used to either discover them or gauge their potencies and breadth.

## 4. Assessing Breadth and Potency of Antibodies

This section of the review provides a brief background to the assays used firstly to identify HIV monoclonal antibodies and secondly to measure the neutralization breadth and potency of antibodies elicited by infected individuals. Initially, phage display combinatorial libraries were used to identify monoclonal antibodies in HIV-infected individuals [94]. To determine the potency and neutralization breadth of HIV monoclonal antibodies, peripheral blood mononuclear cells (PBMCs) were used for the neutralization assays. The PBMC assays have known inherent technical problems, such as donor cell variability, which likely produce differential neutralization profiles, potencies, and susceptibilities to infection with different reference strain viruses. Consequently, this impacted on the comparability of data between laboratories, which also brought into question the interpretation of antibody neutralization breadth and potency. The TZM-bl cell line, formerly JC53-bl is a HeLa cell line, that expresses CD4 and CCR5 receptors, was generated by introducing separate integrated copies of the luciferase and β-galactosidase genes under control of the HIV-1 promoter [95]. The TZM-bl cell line is used in a microneutralization assay format to measure the antibody-mediated neutralization of HIV-1 as a function of reductions in HIV-1 Tat-regulated firefly luciferase reporter gene expression after a single round of infection with Env-pseudotyped viruses. This assay, which replaced the somewhat cumbersome PBMC assays, paved a way forward for the field, and allowed for laboratories across the world to compare their data [96,97,98]. As a result, from the mid-2000’s, the discovery of the broad and potent antibodies was radically different. The advent of single memory B-cell technology and cloning further catapulted the field into a new era and revolutionized the discovery of a range of potent and BnAbs [99,100,101,102,103,104,105,106,107]. Newer, broader, and potent antibodies were discovered from the sera of HIV-infected individuals, paving a pathway for these antibodies to be tested as proof of concept in preventing HIV through passive transfer.

Passive transfer of BnAbs in murine [108,109,110,111,112] and preclinical non-human primate studies have shown promising outcomes, with many conferring complete protection upon systemic or mucosal viral challenge including oral, intravaginal and intrarectal [104,113,114,115,116,117,118,119,120,121,122,123,124]. In addition, these antibodies also provided a blueprint/clues for HIV vaccine development to reverse engineer immunogens sequenced and mapped from their cognate epitope binding sites on the virus, to stimulate and coax the immune system into producing such antibodies via vaccination. This section of the review focuses primarily on recent BnAbs that have moved into the field, not only providing clues for vaccine development, but paving the way for passive immunity using these BnAbs as pre-exposure prophylaxis (PrEP).

## 5. Early Clinical Trials and Recent Testing of Passively Administered BnAbs

Certain landmark and early passive immunity studies in HIV-infected humans that have set precedence using BnAbs should also be mentioned. With the discovery of some of the earliest monoclonal antibodies from HIV-infected individuals that displayed neutralization-b12, 2F5, 4E10, and 2G12 [94,125,126,127,128] in the 1990’s and early 2000’s, there was reinvigorated interest in pursuing passive immunity as a therapeutic and prevention option. These studies mainly tested the safety of BnAbs in HIV-infected individuals using 2F5, 2G12 [129,130] and 4E10 [131,132] discovered in subtype B infected long-term non-progressors. Besides these antibodies demonstrating safety, these early phases I studies also demonstrated the importance of using an antibody cocktail or combination due to the emergence of resistant viruses in HIV infected individuals receiving these BnAbs. Additionally, these studies highlighted the importance of viral sequencing to understand the emergence of resistant viruses during passive immunization using monoclonal antibodies as a means of viral control in HIV-infected individuals [133]. Although deemed safe for use in HIV-infected individuals, trials testing these early BnAbs did not proceed to HIV prevention in healthy individuals. Part of the reason was that there were in vitro data for 2F5 and 4E10 [134], exhibiting phospholipid and cardiolipin binding activity [135], similar to those of autoimmune anti-cardiolipin antibodies which lead to immune complex formation and thrombosis. Recently, another antibody 10E8 [100], although shown to be highly potent, also raised safety concerns due to the local reactogenicity it caused in early phase I studies. All of these antibodies-2F5, 4E10 and 10E8–belong to a class of antibodies that target the membrane proximal external region (MPER) of the virus. These MPER antibodies act similarly to fusion inhibitors, where they interact between the viral and target cell membrane, occluding the binding of the viral and target cell membranes interface. The affinity and non-specific binding of the MPER class antibodies for cell membranes is therefore unsurprising. Considering the safety concerns, the use of these combinations of BnAbs that included an MPER antibody were put on hold.

Since the early passive immunity studies in humans, there has been an explosion in the discovery of potent BnAbs. Several of these BnAbs have been pursued and tested as proof-of-concept studies for HIV prevention in passive immunity trials. VRC01, for example, is a CD4 binding site antibody that has been tested in two Phase IIb trials in high-risk women (HVTN 703), MSM and transgender (HVTN704) populations as an intravenous infusion [105,136,137]. VRC01 was tested in preclinical macaque studies where it achieved 75% efficacy [122,138,139]; furthermore, VRC01 did not significantly reduce overall HIV acquisition in either of these antibodies mediated prevention (AMP) trials. Both AMP trials showed that intravenous administration of VRC01 at eight-week intervals over twenty months did not significantly reduce HIV transmission overall. These findings are important given that the in vitro sensitivity studies for eighty weeks of the trial showed 75% prevention efficacy at a median IC80 of <1 μg/mL against the susceptible HIV strains tested in sub-Saharan Africa, South America, Switzerland, and the United States. There was no prevention efficacy in the intermediate or resistant viruses at a median IC80 of 1–3 μg/mL and >3 μg/mL of VRC01. In the trials, only 30% of the HIV strains circulating were susceptible to VRC01, rendering the resistant HIV strains able to escape VRC01 neutralization resulting in breakthrough infections [137]. Irrespective, the gender, subtype, population, dosage, or route of HIV acquisition did not impact on the prevention efficacy. These data further highlight the extraordinary diversity of circulating HIV strains and argues for the use of a cocktail of BnAbs with multiple specificities. The rationale is to improve coverage, targeting different conserved and discontinuous epitopes on the viral envelope, and to prevent infection by viral resistant strains [140,141,142].

Other clinically advanced BnAbs undergoing trial testing for prevention and treatment include 3BNC117 [143,144], VRC07–523 and their variants that target the CD4 binding site (CD4bs) [139], N6 [99]; 10-1074 [101], and PGT121 [104] which recognize the base of the V3 loop and surrounding glycans; 10E8 [100,145] which binds to the membrane proximal region (MPER); PGDM1400 [103], and CAP256 [146] which recognize the V1–V2 loops and associated glycans. Currently, Phase I clinical trials in South Africa at the Centre for the AIDS Programme of Research in South Africa (CAPRISA) are testing three of the nine BnAbs and includes engineered BnAbs variants VRC07–523LS, CAP256V2LS and PGT121. These BnAbs are either administered subcutaneously or intravenously as a single or a combination of these BnAbs [147,148]. The results from these trials are expected later in 2021. The advantage of a combination cocktail of BnAbs is that they may work together synergistically and/or additively to increase their effectiveness against circulating transmitted viruses [149]. With current treatment guidelines of immediate ARV initiation in HIV infected individuals, the discovery of newer and more potent BnAbs will likely be hampered. In a minority of infected individuals, breadth, and potency of BnAbs develop over time. In the absence of ARV treatment, this process is driven by ongoing viral antigen stimulation. Ongoing antigen stimulation then drives somatic hypermutations [150] and affinity maturation [151] which then fine tune the antibodies to ultimately developing breadth and potency. While clinical testing of combination BnAbs in high-risk populations is attractive, the feasibility and sustainability of such an approach would be a major obstacle to its long-term application. Therefore, the development of a vaccine capable of eliciting the production of BnAbs remains one of the major goals of HIV vaccine development.

## 6. Current Vaccine Candidate Pipeline to Stimulate the Production of BnAbs

While we recognize that passive immunization with BnAbs is not a long-term solution to HIV prevention, other alternative vaccine strategies have been emerging. One of the strategies has involved testing the concept of sequential vaccination in humans [152] as a means of coaxing the immune system towards developing BnAbs to HIV, termed as “reverse vaccinology”. The underlying tenet for this approach is stimulating naïve B-cells with the right immunogen to drive the process towards developing these BnAbs. Sequential immunization involves a series of engineered proteins through germline targeting to prime BnAb precursors. This concept was tested earlier in various animal studies [153,154] and genetically modified knock-in mice, using a protein nanoparticle, eOD-GT8 60 mer [155]. eOD-GT8 60 mer, based on VRC01 CD4-binding site, was engineered to activate the B-cell precursors that are able to elicit BnAbs. This animal study confirmed that these humoral immune responses can be elicited through sequential immunization and steer the immune system towards producing BnAbs. The natural progression was to test this concept in humans, and the first phase I, placebo-controlled clinical trial testing the safety of eOD-GT8 60 mer with AS01B adjuvant was initiated in 2018 in 48 healthy individuals. Recently, in the first quarter of 2021, scientists at the 2021 R4P Conference revealed promising preliminary findings that 97% of participants who received two-dose priming of eOD-GT8 60 mer immunogen candidate developed precursor VRC01-class IgG B cells, to broadly neutralizing VRC01-class antibodies [156]. These data are very encouraging and confirm, at least in part, that stimulating such rare B-cell precursors in humans is possible, and thus paves a pathway for such types of vaccines not only for HIV but other infectious diseases. The litmus test for success of reverse vaccinology or sequential immunization with subsequent boosting is to assess the epitope-specific single B-cell responses through single cell sorting and deep sequencing of the B-cell receptors. These preliminary data would be important to understand whether this approach works consistently, that the right B-cells are being primed. Assessing the development and evolution of memory B and plasma cells capable of eliciting the production of BnAbs, would be central to understanding if durable humoral immunity using sequential immunization is possible. Recently, mice studies have shown that this may indeed be possible [157]. However, the feasibility and practicality of sequential immunization will pose challenges in real world settings. Furthermore, the complexity of eliciting BnAbs cannot be overstated. By their very nature, B-cells that produce BnAbs during HIV infection have very long third heavy chain (HC) complementarity determining regions (CDRH3s); during this process, these B-cells would naturally undergo immunological checkpoints and are often deleted very early on [158,159]. Another caveat is that these B-cells that can elicit BnAbs also have to undergo a series of high-level somatic hypermutations and affinity maturation [158,159]. Importantly, dissimilar to T-cells, B-cells not only undergo clonal expansion, but during the clonal expansion process, the B-cell antibody gene further diversifies through mutations, leading to honed antigen specificity and higher affinity [160,161,162,163]. In addition, in the case of B-cells that produce these BnAbs, if the mutations are removed these germline reverted antibodies lose both their affinity and their neutralizing ability [102,164,165]. The data underscore the immense complexity of B-cell development and their ability to produce antibodies that are functionally effective. It is expected that the immunogens used for sequential immunization and boosting to produce BnAbs will mimic the very process that occurs during natural infection.

Besides trying to coax the immune system with the apt protein/immunogen conformations for immune system recognition to develop BnAbs, alternative strategies using a recombinant vector with genes encoding BnAbs are available. Recombinant adeno-associated virus (rAAV)-mediated delivery of HIV BnAbs genes show a promising alternative strategy for a preventive HIV vaccine. A study performed using a humanized mouse receiving rAAV vector delivering genes encoding for VRCO1, b12, 4E10, 2F5 and 2G12 antibodies conferred full protection from HIV infection, even with high-dose intravenous HIV challenges [166]. Several other studies have also shown the antiviral efficacy of BnAbs, such as PGT121 and CAP256.VRC26.25, delivered by using rAAV in HIV-infected humanized mice [167,168]. Rhesus macaques receiving the AAV gene-encoded BnAbs for 3BNC117, 10-1074 and 10E8 post-infection with SHIV experienced abrupt and significant declines in plasma viral loads, and these animals had undetectable viraemia for 3 years [169]. However, the positive results gained from these studies was short-lived, as anti-drug antibody (ADA) responses later developed, undermining the successes achieved from the expression of antibodies from rAAV vectors. Similarly, in NHPs receiving rAAV8 vector, encoding the gene for the simian version of the VRCO7 BnAb, NHPs had protection from mucosal SHIV infection for less than one month after vector delivery. There was a corresponding decline in the concentration of VRCO7 to undetectable levels due to ADA response [123]. Currently, a Phase 1 clinical trial evaluating rAAV8-vector expressing VRCO7 is underway in HIV-1-infected adults with controlled viremia [170]. Although this vectored approach holds promises in HIV prevention and cure, further studies are required to improve rAAV delivered BnAbs to mitigate ADA responses which can ultimately undermine such technology.

Together, these studies underscore the incremental strides made in the HIV vaccine field and reminds us of the challenges in developing an effective vaccine. How effective these elicited humoral responses are through either active vaccination or passive immunization in vulnerable mucosal surfaces, such as the genital tract or rectal surfaces, remains to be fully defined.

## 7. Mucosal Immunity

The majority of HIV infections occur through sexual transmission and involves the interaction of the virus with mucosal epithelium [171]. Studies conducted on rhesus macaques, documenting the initial stages of infection of the genital tract tissue after vaginal SHIV inoculation, shows that the virus can rapidly form foci in genital tissue. Thereafter, viral dissemination occurs in the lymph nodes and the constant seeding of the infections to lymph nodes during the systemic spread was from the initial site of small founder populations of virus-infected cells that formed these infection foci [172,173,174]. Therefore, it is imperative that HIV vaccines confer protection at the portals of entry, such as the genital or rectal surfaces, to protect against sexual transmission. The induction of BnAbs through vaccination is regarded as the holy grail in preventing HIV infection and, to date, a vaccine able to stimulate robust and durable antibodies is yet to be achieved [175,176]. Whether parenteral vaccination and or direct mucosal vaccination can confer partial or sterilizing immunity remains largely unknown. The genital tract displays several unique features that are not common to other mucosal surfaces, such as the rectum, highlighting the challenges in developing a vaccine that can protect at least across these two diverse mucosal surfaces.

### 7.1. Structure of the Vaginal and Rectum Mucosa

Within the female genital tract (FGT) alone, there is compartmentalization and distinct microenvironments with diverse immunity. The FGT is comprised of the vagina, ectocervical and endocervical regions. The ectocervical and vaginal mucosa is lined by non-keratinized stratified squamous epithelia, with a larger proportion of Langerhans cells (LC) in the ectocervix than in the vagina. In contrast, the endocervix and upper reproductive tract are lined by simple columnar epithelial cells lacking LCs [177]. The connective tissue layer, known as the lamina propria, beneath the epithelial layer of THE FGT contains B cells, CD4+ and CD8+ T-cells and antigen presenting cells (APCs) that form the inductive and effector immune sites [178]. The vaginal mucosa lacks secondary lymphoid tissues as an inductive mucosal site, therefore the priming of adaptive response occurs in draining the lymph node. Intraepithelial γδ T-cells, macrophages, LCs and submucosal dendritic cells (DCs) in the vaginal canal and natural killer (NK) cells and regulatory T-cells in the uterus play important roles in protection against virus [179]. Another major mucosal HIV transmission route is through rectal mucosa, especially in MSM populations, although the contribution of HIV transmission through anal sex in heterosexual couples remains largely undefined. The rectal mucosa is very fragile and is lined by a single-layer columnar epithelium containing lymphoid nodules that are similar to Peyer’s patches and large numbers of macrophages and few numbers of DCs. Mucosal tissue of rectum also contains abundant microfold cells (M-cells), which transport antigen across the epithelial barrier and generate immune responses [180,181].

### 7.2. Innate and Adaptive Immune Responses

Early innate immune responses against invading pathogens at the mucosal surfaces include epithelial barrier mucus, pH, antimicrobial, and anti-inflammatory peptides [182]. Other components of innate immune response involve pattern recognition receptors (PRR) such as Toll-like receptors (TLRs) on the epithelial cells. The binding of microbial components to TLRs trigger the release of cytokines and chemokines, sending signals to immune cells such as macrophages, DCs, neutrophils and NK cells. Macrophages are phagocytic cells, that can internalize antigens and destroy the pathogen by lysosomal degradation. Macrophages are irregularly distributed in the FGT, with more concentrated in the endometrial stroma and myometrial connective tissue [183]. Macrophages in the vagina express higher levels of CD4 receptors and coreceptors, CCR5 and CXCR4, making them highly susceptible to HIV infection [184,185]. DCs in the vagina are mostly found within the epithelial layer. Various subsets of DCs, expressing different lineage markers, are present in the FGT [186,187,188,189,190]. CD103+-expressing DCs were found exclusively in the endometrium, CD14- and DC-SIGN-expressing DCs in the cervix and CD1a+ DCs in the vagina [188,191]. A study demonstrated that DCs in the FGT on HIV exposure can secrete CCR5 ligands, chemokines and antimicrobial elafin and SLPI with no change in the proinflammatory molecules [188], suggesting that DCs in the FGT not only capture and disseminate the virus but also induce antiviral responses and recruit HIV target cells. Therefore, further studies understanding the diversity of DCs in the FGT, and their role in HIV acquisition and protection, is needed. Neutrophils constitute 10–20% of the immune cells in the FGT. Neutrophil plays a very crucial role in the innate immune response through various mechanisms such as ROS release, phagocytosis, degranulation, and neutrophil extracellular trap (NET) formation [192]. A recent in vitro study demonstrated that genital neutrophils are able to inactivate HIV virus by entrapment in the NETs [193]. In addition, clinical studies demonstrated associations between low neutrophil count and increased HIV risk in women [194,195]. In contrast, other studies showed increased neutrophil recruitment with STIs and genital inflammation, leading to increased release of innate molecules such as defensins and proteases increasing HIV susceptibility in the FGT [196,197,198]. Therefore, the potential role of neutrophils in HIV infection under these pathological conditions needs to be further investigated. NK-cells in the FGT possess distinct phenotype/s compared to blood NK cells. NK-cells play an integral part in the innate immune response, consisting of 70% of leukocytes in the endometrium and mediate cytotoxic functions through Fc-receptor (FcR), expressed by most NK-cell subsets [199]. NK-cells in the uterus produce proinflammatory cytokines and chemokines to activate macrophages and generate the cytotoxic T-cell [200].

Failure of the first line of protection results in the induction of antigen specific adaptive immune responses driven by T-cell responses following activation by APCs or B-cells producing antibodies. Cell-mediated immunity is driven by effector CD8+ T-cells upon activation, induces apoptosis and granzyme-mediated cytolysis [201]. CD4+ T-cells are sub-divided into Th1, Th2, and Th17 and T-regulatory cells (Treg) cells, according to their effector cytokines. A study performed in mice showed CD4+ T-cells can control the migration of effector CD8+ T-cells by secretion of IFNγ, that mediates the destruction of the virus in the vaginal tissues [202]. This is an important consideration for the induction of effective cellular immune responses in the FGT. In the female reproductive tract, CD8+ T-cells are more frequent than CD4+ T-cells [203,204]. Furthermore, lymphocytes aggregates (LA) in FGT consists of a B-cell core predominantly surrounded by CD8+ T-cell, which in turn is encapsulated with macrophages. The composition of LA and its activity in the FGT is regulated by the hormones during the menstrual cycle [205,206]. In the uterus, during the secretory phase, cellular immunity is suppressed following the ovulation, thus impacting HIV susceptibility. However, cellular immunity persists throughout the menstrual cycle in the lower reproductive tract [207]. This further highlights the importance of understanding the hormone-mediated changes in the FGT for the successful development of mucosal vaccines. Th17-cells are abundantly present at the mucosal surface compared to other T-cell subsets and maintain mucosal barrier integrity [208] and respond to bacterial and fungal pathogens [209]. Th17-cells are also shown to regulate neutrophil recruitment and migration in the FGT [210]. T-regs, on the other hand, prevent inflammation in the mucosal tissues thereby maintaining the balance for optimal and effective functioning of the immune system.

Humoral responses in the FGT are distinct from other mucosal surfaces. Dissimilar to other mucosal surfaces where IgA is the major antibody isotype, in both the FGT and male genital tract (MGT), IgG is a more dominant isotype than IgA and a lower proportion of IgM are present [211]. Polymeric immunoglobulin receptor (pIgR) transport secretory IgA (sIgA) across the epithelial cells into the lumen to prevent mucosal invasion [212]. Expression of pIgR in the epithelium of the FGT is affected by hormones that results in a difference in the antibody concentrations during the menstrual cycle [213]. Furthermore, there are two subclasses of IgA: IgA1 and IgA2. The FGT contains a higher proportion of IgA2, whereas IgA1 dominate in the semen s [214]. IgG found in the mucosal tissues is derived from local IgG-secreting plasma cells or from the blood. Specialized IgG-specific neonatal Fc receptors (FcRn) expressed in the epithelial cells can mediate IgG bidirectionally across the epithelial barrier. FcRn binds IgG in a pH-dependent manner with the binding of IgG at an acidic pH and release of IgG at a neutral pH [215]. Antibodies represent the principle correlate of protection against HIV and can have both neutralizing and non-neutralizing functions. Neutralizing antibody functions are mediated by the antigen-binding fragment (Fab), non-neutralizing effector functions are mediated by binding of antibody’s fragment crystallizable (Fc) region with Fc receptors (FcR) on immune cells. Various non-neutralizing antibody functions include ADCC, antibody-dependent cellular phagocytosis (ADCP), and also complement activation. Many studies have shown the potential protective role of neutralizing antibodies in both animal and human models [216]. However, in the RV144 vaccine trial, IgG antibodies correlated with lower HIV-1 risk and were able to drive ADCC, ADCP and complement activation [79,217], underscoring the role of non-neutralizing effector functions in protective vaccination. In a recent study, HIV-specific antibodies demonstrated antibody-dependent neutrophil phagocytosis (ADNP) and ADCC functions in the genital tract [218]. In addition, HIV-specific ADCC directly and significantly correlated between the blood and genital compartments. However, further studies are required to explore the role of IgA- and IgG-mediated effector functions in the genital tract.

Cytokines and chemokines play an important role in regulating both innate and adaptive immune responses in the FGT. Binding of microbial components with TLRs expressed on innate immune cells induce a series of cascade pathways secreting cytokines and chemokines. The cytokines influence the development of different CD4+ T-cell subsets, promoting cellular and humoral responses [219]. Pro-inflammatory cytokines such as IL-12, IL-23 and TGF-β regulate differentiation of CD4+ T-cell subsets that promotes different antibody responses [219]. Several studies have shown that Th1-cells can promote IgG whereas Th17-cells induce IgA responses at the mucosal site [220,221,222,223]. Vaccines promoting inflammatory responses may alter systemic Th-1-mediated IgG response to Th-17 cells and IgA responses when delivered at mucosal site [224,225,226]. Thus, the administration of the same antigen and adjuvant may produce different immune responses based on the route of administration. A combination strategy inducing effective innate and adaptive responses that can act at the portal of entry will be necessary for the development of successful mucosal vaccines.

Interestingly, the expression of “homing receptors” or integrins on B- and T-cell and chemokines that promote cell migration controls the cross-talk between different mucosal sites, playing an important role in the mucosal immunity [227,228,229]. IgA-secreting B-cells expressing homing receptors α4β7-integrin, after activation in intestinal inductive sites, leave through the lymphatic system into the bloodstream, resulting in homing to the intestinal mucosa [230]. The importance of homing receptors is an important consideration in vaccine development. Mucosal immunization can induce antibody responses in the systemic circulation due to the presence of α4β1-integrin and leukocyte (L)-selectin peripheral homing receptors on the B-cells activated in the mucosa [230]. In contrast, B-cells activated in the peripheral lymph node after systemic immunization are unable to induce mucosal antibody responses due to the absence of mucosal homing receptors. The aforementioned data provides evidence that mucosal immunization can generate both mucosal and systemic immune responses by inducing the homing receptor. The events that occur at the genital mucosa, the portal of entry, play an important role in HIV transmission and provide opportunity for intervention for future research.

### 7.3. Vaginal and Rectal HIV Transmission

Both cell-free HIV virions or cell-associated HIV can traverse the epithelium through transcytosis or gaps between epithelial cells to infect the target cells such as CD4+ T-cells in the lamina propria [231]. Once HIV enters the lamina propria, it causes he depletion of CD4+ T-cells. Alternatively, in the absence of organized lymphoid tissues in the FGT, intraepithelial and sub-epithelial DCs take up the antigen, then mature and migrate through the lymphatic system to the draining lymph nodes in order to prime naïve T-cells [175]. Phenotypic analysis revealed that most HIV infections through sexual intercourse occurs in the CD4+ T-cells expressing CCR5 co-receptors, indicating the significance of the R5 phenotype of the transmitted virus [232]. However, the FGT also contains a large population of CD4+ T-cells expressing CXCR4 co-receptors. HIV susceptibilities vary with different subtypes of CD4+ T-cells, such as T-cells expressing α_4_β_7_ integrin, a gut homing receptors is highly susceptible to HIV infection [233]. HIV-1 gp120 have been shown to bind α_4_β_7_ integrin receptors to facilitate mucosal transmission, showing the role of this homing receptor in HIV infection. Human and animal studies showed the depletion of Th17-cells in the FGT and the gut during HIV infection [234,235,236], indicating both preference and susceptibility to HIV infection. Th17-cells co-expressing CCR5 and CD90 were also highly susceptible to HIV infection in the FGT [237]. In macaques, Th17-cells are identified as the primary target of SIV infection during vaginal transmission [238]. Therefore, the loss of Th17-cells during HIV infection compromises the mucosal epithelial barrier integrity, which further exacerbates inflammation and results in viral replication and dissemination [239].

The role of T-regs in HIV susceptibility can also be double edged-sword. T-regs can be beneficial in decreasing the immune activation and inflammation thereby limiting the target cells for the virus, or detrimental, by inhibiting specific HIV immune responses through suppressive potential and thus promoting viral persistence [240,241,242]. T-regs in the FGT showed a high expression of CCR5 which not only increases their susceptibility to HIV infection but also fuels HIV replication [243] and likely the formation of infection foci in the mucosal tissue. Understanding the differential infection of these T-cell phenotypes in HIV susceptibility in the FGT allows for new opportunities in enhancing vaccine efficacy and other preventative interventions. In the rectal mucosa HIV entry is more efficient than the FGT due to the single layer of columnar epithelia, which upon disruption allows easier viral entry and access to HIV target cells in the lamina propria [244]. In addition, M-cells in the mucosal tissue of the rectum provide a rapid pathway for HIV entry across the epithelial barrier [175,245]. Design and implementation of efficacious mucosal vaccines that interfere with viral transmission require an in-depth understanding of the initial infection events in the mucosa. The transmission rate in the female partners of infected males is significantly more efficient than female-to-male transmission [246]. Presence of SIV/HIV have been shown in several male genital organs, contributing to viral load in the semen during the early and chronic infection stages [247]. Both the male genital tract and the FGT have unique immunological milieu, characterized by diverse physiological barriers and unique innate and adaptive immune responses, regulated by hormonal changes. Therefore, a comprehensive investigation of HIV transmission in both the FGT and the MGT is required.

Another important aspect to consider in the HIV transmission at the mucosal surfaces is cell-associated HIV-1 mucosal transmission. Both cell-free virions and cell--associated HIV have been found in genital secretions which lead to sexual transmission [248], although their individual or combined contribution to HIV acquisition remains unknown. Studies performed in human and macaque mucosal explants showed cell-associated HIV transmission to be more efficient than by cell-free virions in male, female, and anorectal mucosa [248,249,250,251,252,253]. In the FGT, vaginal microbiota and STIs can influence cell-associated HIV transmission [254]. High efficiency of cell-to-cell infection may be partly responsible for the failure of several recent vaccines and microbicides in preventing HIV transmission in the mucosal compartment [255]. However, few studies addressed the importance of mucosal cell-associated HIV transmission [254], and therefore, this warrants further research for the development of vaccines strategies to prevent HIV infection.

Studies have shown that regardless of the route of HIV transmission, whether mucosal or systemic, HIV predominantly replicates in the mucosal tissues that are rich in target CD4+ T-cells [175,256,257]. Therefore, efforts are being made to develop vaccines against HIV that are effective at the portals of viral entry, the genital tract or rectum, and prevent the establishment of viral reservoirs. In the next section of this review, we will discuss current approaches to the design and development of vaccines that can protect against genital or rectal mucosal transmission of HIV.

## 8. Strategies for Mucosal Vaccine Development

New mucosal vaccines with pattern recognition receptors (PRRs) agonists as adjuvants can be advantageous in generating effective immune responses in the FGT. Hence, the synergistic effect of the PRR agonist may be warranted in mucosal vaccine strategies. Toll-like receptors (TLRs), upon activation through the cognate ligand binding on innate immune cells such as dendritic cells or macrophages, can trigger intracellular signaling pathways leading to immune activation and cytokine secretion which in turn stimulates adaptive immune responses [258]. Many studies have tested the efficacy of TLR ligands as adjuvants in parenterally administered HIV and SIV antigens in human and NHP; however, very limited studies have been pursued in exploring mucosally administered antigens in conjunction with TLR ligands [259]. Vaginal immunization with immunogen tetanus toxoid co-administered with the TLR2/6 agonist, FSL-1, improved the vaginal IgA response in mice [260]. However, the TLR2/1 agonist failed as a promising mucosal adjuvant for inducing humoral immune response in macaques [261]. A potential caveat in using the PRR approach, including TLR agonists as adjuvants, may be an increase in HIV infections resulting from increased homing of mucosal CD4 T-cells and subsequent immune activation of these target cells in the genital tract [262,263,264,265,266]. In an in vitro study, TLR2 ligand binding to TLR2 expressed on human CD4+ T-cells from blood resulted in up-regulation of CCR5 expression, proliferation, and enhanced HIV replication, increasing susceptibility to HIV infection. As a result, the TLR2 agonist may not be a desirable candidate for adjuvants for HIV vaccines [263,264,265,266]. In contrast, the TLR3 agonist was demonstrated as a potential adjuvant to enhance mucosal and systemic antibody responses to HIV gp140 and other immunogens in an animal study [260]. Rectal administration of the TLR3 ligands polyinosinic-polycytidylic acid (pIC) analogue PICLC, before or during rectal SIV challenge can limit SIV infection [267]. Nonetheless, further studies are warranted to test the potential role of the TLR3 agonist as a mucosal adjuvant for HIV vaccines. Administering HIV gp40 protein with the TLR7/8 agonist, imiquimod (R848) vaccine using vaginal rings in sheep, demonstrated enhanced antibody production detected in the vaginal secretions [268]. Similarly, rhesus macaques administered with a SIV vaccine with a TLR adjuvant produced Env-specific antibody responses in the vagina [269]. The TLR7 agonist, vesatolimod (GS-9620) combined with V3 glycan dependent BnAbs PGT121, administered after ART discontinuation in SHIV infected rhesus monkeys delayed viral rebound [270]. Mice immunized with gp140, in combination with two TLR9 agonists, alpha-galactosylceramide (αGalCer) and CpG-oligodeoxynucleotide (CpG-ODN), enhanced both cellular and humoral responses in the vagina [271]. However, TLRs may respond differently in different mucosal compartments. Therefore, the results of vaccine administration studies testing TLR agonists as adjuvants may vary across different mucosal tissues. Further studies are required to determine whether TLR agonists as a mucosal adjuvant can achieve similar results in humans. Taken together, these studies demonstrate the importance of adjuvants that need to be considered in the design of mucosal vaccine strategies.

Besides TLR agonists, cytokines are also key molecules that can enhance the innate and adaptive immune responses, resulting in promising vaccine adjuvants. Fusion protein IL-2/Ig, consisting of interleukin-2 (IL-2) and Fc portion of immunoglobulin G (IgG) or a plasmid encoding IL-2/Ig with DNA vaccines, are able to induce immune responses reducing viral replication and disease progression after intravenous SHIV infection in rhesus monkeys [272,273]. Other cytokines and chemokines that have been tested in mice as mucosal adjuvants include IL-1, IL-5, IL-6. IL-12, IL-15, IFN and RANTES monocyte chemoattractant protein 1, macrophage inflammatory protein 1α and MIP-1β [179]. Intranasal and intramuscular DNA vaccination with plasmid encoding RANTES induced humoral and cell-mediated immunity [274]. Intramuscular injection of SIVmac239 DNA vaccines, together with Th-1 promoting IL-12 as adjuvant, developed increased SIV-specific cellular immune responses [275]. However, whether mucosal immunization can generate similar responses in humans and NHP is unknown. In addition, increased HIV-1 specific antibodies were observed in the serum and mucosal compartments of macaques primed by interferon-induced protein (IP-10) adjuvant with HIV-1 DNA followed by a monophosphoryl lipid A and QS-21 adjuvated Env protein boost [276]. The potential of cytokine adjuvants in the HIV vaccine in inducing protective immune responses in the mucosal compartment of the human still needs to be explored to develop effective HIV vaccines. Cytokines stimulating B-cells such as thymic stromal lymphopoietin (TSLP), a proliferation-inducing ligand (APRIL) and B-cell-activating factor (BAFF), which are strong inducers of humoral responses, have been tested as mucosal adjuvants in several studies [277,278]. Intranasal immunization in mice, using TSLP but not APRIL or BAFF as adjuvants, demonstrated strong humoral responses to HIV gp140 in the serum and mucosa [277]. Further studies are required to determine the role of TSLP in inducing mucosal immune response in humans. Immunization of mice with recombinant DNA plasmid encoding BAFF and APRIL, together with IL-12 and HIV-1 Env gp140, induced neutralizing antibodies in the blood [279]. In a recent study, a fusion construct of HIV-gp 140 with APRIL, BAFF and CD40L enhanced HIV-1-specific antibody responses in the vaginal lavage of mice [280]. These findings suggest that APRIL and BAFF could be further explored as mucosal adjuvants for HIV vaccines.

An effective mucosal anti-HIV vaccine should be able to generate multiple immune responses, including cell-mediated immune response and HIV-specific antibodies in the mucosal compartments and in the circulation. Several studies have shown that cell-mediated immunity following mucosal vaccination has significantly contributed to reducing plasma viral load [281,282,283,284]. Intrarectal vaccination of macaques with SHIV and HIV peptide vaccine induced specific cytotoxic T lymphocytes (CTLs), resulting in reduced viral titers to undetectable levels in both blood and the intestine, compared to the same vaccine given subcutaneously [281]. Immunization of rhesus macaques with attenuated SHIV 89.6, induced antiviral CD8+ T-cells in the genital tract provided protection against vaginal SIV challenge [285]. Similarly, rhesus cytomegalovirus (RhCMV) vectors established effector memory T-cells that associated with SIV control in the FGT [45]. HIV-specific antibodies at the mucosal surface can neutralize the virus by providing an additional layer of protection. Local production of anti-Env IgG antibodies, especially against gp41 in the FGT in response to SIVmac239Δnef vaccination, provided protection against vaginal SIV challenge [286]. Similarly, vaginal administration of the b12-neutralizing antibody against HIV-gp120 protected macaques from vaginal SHIV infection [118]. Interestingly, a novel vaginal ring device delivering CN54gp140 vaccine antigen in the sheep elicited gp140-specific IgA and IgG antibodies in the vaginal secretions [268], providing evidence that local antigenic stimulation can drive HIV-specific humoral immune responses in the mucosa.

In addition to viral neutralization, IgG antibodies locally produced or that transduce from the blood can provide protection in the genital tract through non-neutralizing effector functions, such as ADCP and ADCC [51,287]. Immunization of rhesus macaques with nanoparticle-encapsulated TLR ligand (NP) mixed with virus-like particles form of Env can induce protective mucosal antibody responses with enhanced and persistent antibody dependent phagocytic (ADP) activity [269]. In addition, persistent infection with SIVΔnef elicited Env-specific ADCC is associated with protection against SIVmac239 vaginal and intravenous challenge [51]. These results highlight that in the absence of neutralizing antibodies, vaccine strategies designed to stimulate effector function antibodies may provide protection against HIV infection [51]. However, the potential role of mucosal vaccines in inducing HIV-specific neutralizing or non-neutralizing protective antibodies in the genital mucosa needs to be further investigated. In addition to the direct targeting of HIV-1 by neutralizing antibodies, HIV transcytosis can be blocked across the epithelial barrier in the vagina or the rectum. Thus, vaccines preventing epithelial transmission is highly desirable [288,289,290,291]. Vaccines producing HIV-specific gp120 and gp41 antibodies prevented the binding of HIV to glycolipid, known as galactosylceramide (Gal Cer), which mediates the transcytosis of HIV-1 across the mucosal epithelia [291]. The ability to generate local HIV-specific antibodies using a new delivery system in the FGT has potential application in the future of HIV vaccine development.

Recent progress in understanding the cellular characteristic of Microfold (M) cells and the mechanism of antigen presentation has allowed for the assessment of its role mucosal vaccine development. M-cells are specialized epithelial cells of the mucosal inductive tissues that can transport luminal antigens to the underlying lymphoid tissues to initiate immune responses. Pathogens such as *Salmonella typhimurium*, *Yersinia enterocolitica* and *reoviruses* can bind to M-cells and invade the host tissues [292]. Mice intranasally immunized with HIV-envelope protein encoding plasmid complex to reovirus sigma protein (σ1), which binds to M-cells, were able to induce potent mucosal and systemic HIV-specific antibodies and cytotoxic T lymphocytes (CTL) [292]. Vaccines that can trigger both mucosal and systemic responses are of global importance and therefore an M-cell targeted HIV vaccine could be a useful tool. Furthermore, a study also showed that an HIV vaccine coupled with adjuvants for M-cell ligands can be highly effective in inducing cellular and humoral responses in the mucosal inductive sites [293] and needs further investigation in humans. M-cell targeted HIV vaccines can be a potential approach to improve uptake and efficacy of mucosal vaccines.

## 9. Challenges in Mucosal Vaccines

Mucosal vaccines offer several advantages over parenteral immunization, including their non-invasive application, stimulation of robust systemic and mucosal immune responses and ease of application [294]. Despite these advantages, mucosal vaccines have certain limitations. Mucosal vaccines must overcome several physical and chemical barriers in order to generate strong immune responses. Relatively low rates of absorption of exogenous antigens from mucosal surfaces, following degradation by proteases and nucleases, can affect the vaccine efficacy [295]. Thus, dissimilar to traditional parenteral vaccination, mucosal vaccines may be required in large, repeated doses to induce immune responses. Larger doses can increase the risk of inducing tolerance and thus unable to trigger immune responses [296]. Understanding how these unique features of mucosal surfaces function is the key to design a successful mucosal vaccine.

In addition, the immune milieu within the FGT is differentially affected by the phase of the menstrual cycle which may be challenging for vaginal vaccination [297]. In the mid-follicular phase of the menstrual cycle, vaginal immunization of women with a whole inactivated cholera vaccine containing cholera toxin B subunit (CTB) generated more effective local CTB-specific antibodies compared to women immunized in the mid-luteal phase [297]. The viscous mucus barrier during the luteal phase was suggested as the mechanism that may have inhibited the antigen from binding onto the epithelium, thereby preventing the induction of subsequent immune responses. For direct vaginal vaccine administration, at least, the timing of the menstrual cycle may be crucial and may need to be taken into consideration for some vaccines. The difference in the composition of the IgG and IgA make-up in the FGT and the GIT alone highlights the challenge of a single vaccine’s ability to elicit effective responses across these two very diverse surfaces. Rectal immunization was more effective than oral and vaginal immunization for induction of high level of IgA and IgG in the rectum but unable to generate antibodies in the FGT secretions. Vaginal immunization generated antibodies in the vaginal secretions but failed to generate antibodies in the rectum [297,298,299]. In essence, the likelihood of a one-size-fits-all vaccine that can equally confer protection for these two surfaces alone is unlikely.

Mucosal surfaces are also primed by commensal microbes. Mucosal surfaces are the primary site for local immune cell interactions with microorganisms, their antigens, that can regulate both the innate and adaptive immunity. Therefore, another major challenge that needs to be considered in developing a new mucosal vaccine is to understand the complex interplay between the microbiome and host response to vaccines. Various studies performed in humans and non-human primates have shown the direct effect of microbiomes on humoral immune responses to vaccination [300,301,302]. The relative abundance of microbial species, such as *Lactobacillus* and *Clostridium* IV, in the rectal compartment of rhesus macaques immunized with intradermal HIV-1 DNA vaccine directly correlated with levels of rectal HIV-specific IgA and IgG [303]. In addition, a strong association was observed between the gut microbiome and the parenteral vaccine immunogenicity among the participants in the HIV Vaccine Trails Network 096 clinical trial [304,305]. These data provide further insight into the impact that microbiota can have on the immune responses in humans. Another study showed that the HIV-1 Env vaccine induced CD4+ T- and B-cell responses can originate from a pool of intestinal microbiota (IM) cross-reactive immune cells [306]. These findings suggest that microbiome can shape the repertoire of immune cells and influence the response to vaccine immunogens [306]. Microbiome, in addition to affecting their local milieu, can also impact other sites by translocation of bacterial products such as lipopolysaccharides (LPS) and metabolites [307]. Circulating microbial products are strongly associated with increased immune activation and therefore may impact the developing immune responses following mucosal vaccination. Understanding the crosstalk between the microbiome and the mucosal immune system is also pivotal in developing a successful mucosal vaccine candidate.

## 10. Conclusions

Given the proven efficacy of PrEP, a real challenge for candidate HIV vaccines is testing their efficacies especially in high HIV incidence settings, where PrEP is the standard of care for HIV prevention. Besides the testing of candidate vaccines in the presence of PrEP, the lessons from the past for HIV vaccine development have taught us that a purely empirical approach to stimulating antibody responses does not yield protective immunity. The conformation and design of the vaccine immunogens are critical to the immune system “seeing” the immunogen to develop apt and functionally significant humoral and cell-mediated immune responses. Understanding the landscape of pre-existing immunity to vectorized vaccine delivery systems in target populations is important for vaccine efficacy. B-cell or T-cell elicited vaccine immunity alone is not enough to confer protection; rather, it is clear that both arms of the immune system need to be engaged to prevent infection. To inform on vaccine efficacy and design, in-depth basic science immunological investigations have to be performed in the vaginal and rectal mucosa in the subsets of vaccinees to dissect the nature and function of prevailing mucosal immunity. These data will be crucial to understanding the immune correlates of risk or protection during breakthrough infections and post-vaccination at the portals of virus entry.

A sobering reality with HIV is that during viral transmission, the viral milieu may very likely be heterogeneous and consist of both cell-free and cell-associated virus that permits cell-cell transmission. Irrespective of the HIV prevention method, ARVs as PrEP, induced immunity through parenteral or mucosal vaccination or passively administered BnAbs either as combinations or as bi-specific or tri-specific formulations [308,309], will have to trump both cell-free and cell-cell transmission to be effective in HIV prevention. Early studies of BnAbs revealed inconsistent data regarding the effectiveness of BnAbs during cell-cell transmission [310,311,312]. More recently, heterogeneity of the viruses and the targets of the BnAbs mainly dictated their capacity to inhibit cell-cell transmission [313,314,315]. SHIV infection of macaques using cell-associated virus in the presence of PGT121 demonstrated partial prevention [316], further highlighting the sub-optimal effectiveness of BnAbs in this context. Currently, we understand very little on the transmission dynamics for cell-cell transmission in vivo. Data from in vitro infection model systems suggest that cell-cell HIV transmission is significantly more efficient than cell free virus [317,318]. Successful HIV vaccines would have to overcome all of the converging factors pertinent to the virus: extreme viral diversity, emerging viral resistance, cell-free versus cell-associated virus, and the varied structural conformations of target sites on the virus. Due to biological, anatomical, and environmental differences between and within mucosal compartments, a universal one-size-fits-all approach when it comes to HIV vaccine candidates may be less likely. This is due to vaccine-induced humoral responses likely also having different kinetics and isotype profiles in the FGT versus rectal surfaces for protection against heterosexual and MSM infections. Furthermore, the durability, functional capacity, and effective concentrations of the vaccine-induced immune responses proximal or distal to the site of virus entry will also be critical for HIV prevention.

## Data Availability

Not applicable.
